# Emotion regulation in heavy smokers: experiential, expressive and physiological consequences of cognitive reappraisal

**DOI:** 10.3389/fpsyg.2015.01555

**Published:** 2015-10-13

**Authors:** Lingdan Wu, Markus H. Winkler, Matthias J. Wieser, Marta Andreatta, Yonghui Li, Paul Pauli

**Affiliations:** ^1^Department of Psychology (Biological Psychology, Clinical Psychology and Psychotherapy), University of WürzburgWürzburg, Germany; ^2^Department of Psychology, University of KonstanzKonstanz, Germany; ^3^Institute of Psychology, Chinese Academy of SciencesBeijing, China

**Keywords:** nicotine addiction, smoking, emotion regulation, craving, reappraisal, facial electromyography, late positive potential

## Abstract

Emotion regulation dysfunctions are assumed to contribute to the development of tobacco addiction and relapses among smokers attempting to quit. To further examine this hypothesis, the present study compared heavy smokers with non-smokers (NS) in a reappraisal task. Specifically, we investigated whether non-deprived smokers (NDS) and deprived smokers (DS) differ from non-smokers in cognitive emotion regulation and whether there is an association between the outcome of emotion regulation and the cigarette craving. Sixty-five participants (23 non-smokers, 22 NDS, and 20 DS) were instructed to down-regulate emotions by reappraising negative or positive pictorial scenarios. Self-ratings of valence, arousal, and cigarette craving as well as facial electromyography and electroencephalograph activities were measured. Ratings, facial electromyography, and electroencephalograph data indicated that both NDS and DS performed comparably to nonsmokers in regulating emotional responses via reappraisal, irrespective of the valence of pictorial stimuli. Interestingly, changes in cigarette craving were positively associated with regulation of emotional arousal irrespective of emotional valence. These results suggest that heavy smokers are capable to regulate emotion via deliberate reappraisal and smokers’ cigarette craving is associated with emotional arousal rather than emotional valence. This study provides preliminary support for the therapeutic use of reappraisal to replace maladaptive emotion-regulation strategies in nicotine addicts.

## Introduction

Nicotine addiction is the most prevalent type of drug addiction, and one of the leading causes of preventable diseases (Centers for Disease Control and Prevention, 2011a; World Health Organization, 2011, 2013). Globally, smoking is estimated to kill approximately 6 million people per year with an additional 600000 assumed to be dying from the effects of second-hand smoke (Mathers and Loncar, 2006; Oberg et al., 2011). This sum surpasses even the estimated amount of people killed by HIV/Aids, tuberculosis and malaria combined (World Health Organization, 2012). Smokers are aware of the deadly results of smoking and most of them have tried several times to quit smoking (Al-Yousaf and Karim, 2001; Winickoff et al., 2009; Centers for Disease Control and Prevention, 2011b). However, the majority of them relapse. The relapse rates were reported as high as 75–95% after successful intervention for smoking cessation within 6–12 months (Garvey et al., 1992; Ferguson et al., 2005; Nakajima and Al’absi, 2012).

The social psychological/self-regulation failure view describes nicotine addiction as a cycle of spiraling dysregulation of the mesocorticolimbic dopamine (DA) system (Baumeister and Heatherton, 1996) that plays an important role in reward and motivation (Fibiger and Phillips, 1986). Initial regulation failure sets up impulsive smoking and adds additional negative emotions, until the large-scale breakdown in self-regulation, which results in compulsive smoking (Baumeister and Heatherton, 1996; Bechara, 2005). Supportively, neuroimaging studies implicated that nicotine addicts show abnormal brain functions in prefrontal cortex (PFC; e.g., dorsal medial PFC and both dorsal and ventral lateral PFC) and basal ganglia circuits (Bechara et al., 2001; Lubman et al., 2004; Galvan et al., 2011; Goldstein and Volkow, 2011; Sutherland et al., 2012). These brain regions were also consistently reported to be involved in cognitive emotion regulation (Ochsner et al., 2004; McRae et al., 2010; Mocaiber et al., 2011; Moratti et al., 2011). Overall, this line of evidence may point to emotion regulation deficits in nicotine addicts.

In the field of emotion regulation, cognitive reappraisal has received particular attention. Reappraisal refers to changing one’s interpretation of a situation so as to alter emotion (Gross, 2002). Previous studies have shown that reappraisal is an efficient way to modify emotional responses, including emotional experience, expression, and psychophysiology (Gross, 1998, 2002; Ochsner and Gross, 2005; Gross and Thompson, 2007). Furthermore, compared to other regulation strategies (e.g., suppression, avoidance, drug use) cognitive reappraisal appears to be more effective and more beneficial to long-term physical health (Gross, 1998, 2002; John and Gross, 2004; Ehring et al., 2010).

Previous studies have investigated the relation between nicotine addiction and the use of emotion regulation strategies. The consistent findings are that early smoking initiation, enhanced smoking urges, and failures in smoking abstinence are associated with a more frequent use of maladaptive strategies (e.g., suppression); on the contrary, reduced craving to smoke, greater positive mood, and fewer depressive symptoms are associated with a more frequent use of reappraisal strategies (Fucito et al., 2010; Szasz et al., 2012). Mostly these studies relied on self-reports to investigate the use of emotion regulation strategies and emotional responses. Although self-reports are a valuable source of information about affective experience, emotional reactions are expressed on multiple levels (Lang, 1995). Smokers’ emotional responses such as facial expressions and neuronal correlates as a result of emotion regulation have not been assessed yet.

Therefore, the present study combine multiple measures (e.g., self-reports, psychophysiological measures of facial expressions and neural reactions) to investigate emotion regulation via reappraisal in smokers. Based on previous studies (Bechara et al., 2001; Goldstein and Volkow, 2011; Sutherland et al., 2012), we hypothesized that compared to non-smokers (non-smokers) smokers would show deficits in cognitive emotion regulation via reappraisal. In addition, we assessed the effects of smoking abstinence on cognitive emotion regulation, which has not been studied in previous studies. Some studies have shown that deprived smokers (DS) experience more negative emotions and higher cravings to smoke than non-deprived smokers (NDS), which may contribute to the high-rate of relapse (Cinciripini et al., 2006; Piper and Curtin, 2006). Therefore, we hypothesized that it would be more difficult for DS to regulate emotion as compared to NDS.

Further, most prior work focused on the regulation of negative emotions (Baker et al., 2004; Fucito et al., 2010; Szasz et al., 2012). Little has been known about regulation of positive emotions (with a few exceptions, e.g., Krompinger et al., 2008; Wu et al., 2012). It has been acknowledged that the overall balance of positive and negative emotions predicts subjective well-being (Fredrickson, 2001; Fredrickson et al., 2008). In addition, maladaptive positive emotions (e.g., larger appetitive reactions to smoking cues as compared to non-smokers) have been associated with nicotine addiction (Geier et al., 2000; Winkler et al., 2011). Therefore, the present study aimed to expand previous work by comparing smokers and non-smokers on general emotion regulation competency in the context of both positive and negative stimuli.

Lastly, considering that emotional responses have been widely described on two main dimensions, valence and arousal, it is important to examine how the impact of reappraisal on emotional valence and arousal, is related to cigarette craving in nicotine addicts. Previous studies have indicated that cigarette craving triggers cigarette smoking (Kober et al., 2010a) and cognitive emotion regulation involves neural dynamics parallel to craving regulation (i.e., prefrontal-striatal pathway; Kober et al., 2010b; Tabibnia et al., 2014). In line with this, previous studies have shown that more negative emotions are associated with more cigarette craving (Juliano and Brandon, 2002; Baker et al., 2004; Shiffman and Waters, 2004; Conklin and Perkins, 2005; Bradley et al., 2007; Battista et al., 2008; Nakajima and Al’absi, 2012) and individuals with mood disorders, such as depression and anxiety, are more likely to smoke than normal people (McCabe et al., 2004; Gonzalez et al., 2008; Fucito and Juliano, 2009; Morrell et al., 2010). Accordingly, one may hypothesize that regulating negative emotions might be associated with changes in cigarette craving. However, it is not clear yet whether altering emotional valence and arousal impacts cigarette craving similarly or differently.

To address the above issues, we compared deprived and NDS with non-smokers in general emotion regulation competency. We adopted the reappraisal paradigm in which prior to each emotional stimulus, participants are instructed to regulate emotional responses by reinterpreting the emotional stimulus, e.g., changing the perspective in order to feel less emotion (Hajcak and Nieuwenhuis, 2006; Gross and Thompson, 2007; Ochsner and Gross, 2008; Urry, 2009; Moser et al., 2010; Ray et al., 2010). We used pictorial stimuli from the international affective picture system (IAPS) that has been widely applied in previous studies to assess general emotion regulation competency (Ochsner et al., 2004; Hajcak et al., 2009; Moser et al., 2010). Since emotions are dispositions to action that involve multi-level responses (Lang, 1995), we collected self-ratings (Chae et al., 2008; Robinson et al., 2014), psychophysiological, i.e., facial electromyography (EMG; Geier et al., 2000; Winkler et al., 2011, and brain responses (late positive potential, LPP; Littel and Franken, 2011; Versace et al., 2011) to evaluate emotional changes as a function of cognitive reappraisal. Facial electromyographic (EMG) reactions of the corrugator supercilii and zygomaticus major muscle has been suggested as sensitive index of negative and positive emotions (Dimberg, 1990; Dimberg and Thunberg, 1998; Dimberg et al., 1998; Weyers et al., 2006; Mauss and Robinson, 2009; Wu et al., 2012). The LPP activity is a sensitive index of neural activity to emotional arousing stimuli (Hajcak and Nieuwenhuis, 2006; Hajcak et al., 2009; MacNamara et al., 2009). Therefore, we used EMG activity and LPP activity as well as self-ratings as outcome measures for successful or unsuccessful regulation.

## Materials and Methods

### Participants

Twenty-five non-smokers (12 females) and 50 heavy smokers (25 females), aged between 18 and 53 years, were recruited through Internet advertisements and posters. Participants were pre-screened via phone or email and performed a breath-test in an initial assessment with a portable Smokerlyzer^®^ carbon monoxide (CO) monitor. Persons who smoked an average of at least 10 cigarettes per day during the last 12 months and CO > = 10 ppm were considered as heavy smokers, while persons who had smoked fewer than two cigarettes in their lifetime and CO < = 5 ppm were recruited as non-smokers. Participants who met the criteria for heavy smokers were randomly assigned to one of two groups: the non-deprived smoking (NDS) group (individuals were asked to smoke as normal and to consume one cigarette immediately before they come to the laboratory), and the deprived smoking group (DS; individuals were required to abstain from smoking over-night for about 12 h prior to their lab appointment; Mucha et al., 1999; Stippekohl et al., 2010). All participants had a high school diploma or equivalent, were not taking any prescription drugs, and were fluent German speakers. Extra exclusion criteria included: (1) having a personal history of drug addiction excluding nicotine dependence; (2) having current psychiatric or neurological disorders; (3) currently taking any smoking cessation medications and/or participating in smoking cessation programs. Most participants were students from the University of Würzburg receiving either money (6 euro/h) or course credit. DS were compensated with extra 10 euro for their efforts to abstain from smoking. The study, including all procedures and the consent form, was approved by the ethical committee of the Universities of Wuerzburg and was carried out in accordance with the ethical standards of the fifth revision of the Declaration of Helsinki.

### Materials

In total 125 pictures (25 neutral scenes, 50 positive scenes, and 50 negative scenes) from the IAPS, (Bradley and Lang, 1994; Lang et al., 2005) were used^1^. The three picture categories differed significantly from each other with regard to IAPS normative valence ratings (negative pictures: *M* = 2.82, *SD* = 1.64; neutral pictures: *M* = 5.05, *SD* = 1.21; positive pictures: *M* = 7.28, *SD* = 0.48); positive pictures did not differ from negative pictures on arousal ratings (negative pictures: *M* = 5.71, *SD* = 2.16; neutral pictures: *M* = 2.91, *SD* = 1.93; positive pictures: *M* = 5.71, *SD* = 2.28). The mean difference for valence ratings (or arousal ratings) between positive and neutral pictures was the same as the mean difference valence ratings (or arousal ratings) between negative and neutral pictures. Each picture was displayed at a resolution of 600 pixels × 800 pixels on a computer screen at a viewing distance of 60 cm using Presentation software (Neurobehavioral Systems, Albany, CA, USA).

Auditory instructions (‘maintain’ and ‘decrease’) were recorded in advance. The auditory instructions were presented binaurally via speakers with a sound intensity of 68 dB. All of the neutral pictures were preceded by the ‘maintain’ instruction (i.e., to simply attend to the pictures, allowing themselves to experience whatever feelings happened during picture-viewing) forming a baseline condition. Half of the emotional pictures (i.e., positive and negative pictures) were preceded by the ‘decrease’ instruction (i.e., to reappraise the emotional pictures in order to feel neutral by imagining that the depicted negative or positive scenario would become more positive or more negative, respectively, over time). The other half was preceded by the ‘maintain’ instruction.

Self-Assessment Manikins (SAM; Bradley and Lang, 1994) were used to measure emotional experiences as indexed by self-reported valence and arousal. The SAM is a non-verbal instrument. It consists of five graphic figures representing nine-level ratings for both valence (1 = highly positive, 5 = neutral, 9 = highly negative) and arousal (1 = low arousal, 9 = high arousal). To measure cigarette craving during the experiment, a similar instrument with five bar graphs instead of five graphic figures, developed by Stippekohl et al. (2010), was used to represent nine-level ratings for craving to smoke (1 = low craving, 9 = high craving).

A portable Smokerlyzer^®^ CO monitor (Bedfont Scientific Ltd, Kent, UK) was used to verify the smoking status of the participants. Questionnaires were used to measure the degree of smoking dependence, depressive and anxiety levels via the Fagerström Test for Nicotine Dependence (FTND; Heatherton et al., 1991), the German version of the State Trait Anxiety Inventory (STAI; Laux et al., 1981), and the German version of the Beck Depression Inventory (BDI; Hautzinger et al., 1995).

### Procedure and Apparatus

All experimental sessions were conducted in the afternoon between 12:30 and 7:00 pm. After reading the instructions for the experiment and signing the informed consent, participants completed a CO test and filled in the questionnaires.

Participants were then seated in a comfortable chair in a sound attenuated and dimly lit room. The electroencephalograph (EEG) electrodes and facial EMG electrodes were attached to the scalp and face, respectively. Three initial practice trials were given to explain the procedure. An example of reappraisal was given prior to practice trials, showing participants how to reappraise an emotional picture in order to feel neutral. Participants were asked to speak out aloud how they reappraise pleasant and unpleasant pictures during the initial practice trials. Feedbacks were given till participants completely understood the reappraisal strategy.

Next, the experimental session started, consisting of 125 trials with 25 trials for each of the five experimental conditions (i.e., maintain-neutral, maintain-positive, maintain-negative, decrease-positive, and decrease-negative). The trials were pseudorandomized so that no more than three trials from the same condition were presented successively. Each trial began with a white fixation cross presented on a black screen for a period ranging randomly from 4 to 5 s. The fixation cross turned blue, 1 s before the onset of the auditory instructions (i.e., ‘maintain’ or ‘decrease’) that lasted for about 1 s. Following the instruction, there was a 1 s delay and then the corresponding picture was presented for 6 s. At the offset of each picture, the rating scales appeared on the screen and participants rated how they felt during picture presentation. There were breaks after every 25 trials. The whole experimental session lasted about 40 min.

### Psychophysiological Data Recording

Continuous EMG and EEG were recorded at 1000 Hz by using a V-Amp 16 amplifier (Brain Products Inc., Gilching, Germany). Facial EMG activity was measured over the corrugator and zygomaticus muscle regions according to guidelines provided by Fridlund and Cacioppo (1986). The EEG was recorded using an EasyCap (EasyCap, Hersching, Germany) from 10 positions including FCz, Cz, CPz, Pz, C1, C2, CP1, CP2, and the left and right mastoids. Vertical EOG was recorded from electrodes placed 1 cm above and below the right eye, and horizontal EOG was recorded with two electrodes 1 cm from the outer epicanthus of each eye. FCz was used as ground. Reference was placed at Cz during data recording and replaced by the mean of mastoids during off-line data analysis. Impedance was kept below 10 kΩ at all sites.

### Data Reduction

Off-line analyses of the EMG and EEG activity were conducted with Brain Vision Analyzer Software (Version 2.0, Brain Products Inc., Gilching, Germany). On average 4.51% of the trials were rejected due to Íartifacts, which left an average of 23.87 trials per subject and per condition. Ten participants were excluded from data reduction and further analysis because of technical errors that resulted in a lack of markers in the raw EEG data. As a result, a total of 23 non-smokers (11 males), 22 NDS (10 males) and 20 DS (10 males) were included in data analyses.

Electromyography data were re-referenced to obtain bipolar recordings. The raw signal was filtered with a band-pass filter from 30 to 500 Hz and a 50 Hz notch filter. Subsequently, the data were rectified, smoothed using a 125 ms moving average filter, segmented into trials, and baseline corrected for each trial. Trials with EMG activity above 8 μV or below -8 μV during the baseline (mean EMG activity over 1000 ms preceding picture onset) and above 30 μV or below -30 μV during picture presentation were excluded. EMG activity was scored as the average activity in the time window 300–6000 ms (Dimberg et al., 2000). Before statistical analysis, EMG activity was measured as the difference between the mean activity during the 6 s picture period and the 1 s baseline.

Electroencephalograph data were band-pass filtered between 0.01 and 20 Hz and then segmented into trials (-100–6000 ms with respect to picture onset). Subsequently, the data were corrected for ocular artifacts using the method developed by Gratton et al. (1983). An automated procedure was used to reject remaining artifacts according to the following criteria: a voltage step of more than 50 μV between two sample points, a voltage difference of more than 300 μV within a trial, and a maximum voltage difference of less than 0.50 μV within 100 ms intervals. EEG recordings were then re-referenced to the numeric mean of the mastoids, and baseline (-100–0 ms) corrected. Based on previous research indicating that the LPP is typically starting approximately 300–400ms after stimulus onset (Hajcak and Nieuwenhuis, 2006; Hajcak et al., 2009) and maximal at around 1700 ms at posterior and parietal sites (Schupp et al., 2000; Keil et al., 2002), the LPP was scored as the average activity in the time window 300–6000 ms at CPz, CP1, and CP2. For each participant, self-ratings, EMG and EEG data were averaged across trials per each condition.

### Statistical Analyses

One way analyses of variance (ANOVAs) were conducted to test for differences between non-smokers, NDS, and DS in demographics, degree of smoking dependence, and depression and anxiety levels. To analyze the effect of emotion regulation via reappraisal, difference scores were calculated by subtracting data scores of the baseline condition (i.e., maintain-neutral) from that of the other conditions (i.e., maintain-positive, maintain-negative, decrease-positive, and decrease-negative). These difference scores were then submitted to a repeated measures ANOVA with picture valence (positive, negative) and reappraisal (decrease, maintain) as within-subject factors, and group (NS, NDS, DS) as the between-subjects factor. Dependent variables included self-reported valence, arousal, and craving, corrugator and zygomaticus activity, and the LPP activity. *Post hoc t*-tests were conducted to further examine significant effects. To investigate whether the decreases in emotional feelings are associated with decreases in smokers’ cigarette craving, correlations between changes in cigarette craving and emotional valence and arousal were analyzed on the basis of difference scores calculated by subtracting rating scores under the conditions with ‘decrease’ instructions (decrease-positive, decrease-negative) from corresponding conditions with ‘maintain’ instructions (maintain-positive, maintain-negative). The difference scores were then submitted to Pearson correlation analysis.

For all analyses the alpha-level was set at.05 (two-tailed). The Greenhouse-Geisser correction was applied when the assumption of sphericity was violated. The uncorrected degrees of freedom and effect sizes ($\eta_{p}^{2}$) are reported.

## Results

### Sample Characteristics

The sample characteristics are depicted in **Table 1**. The one way ANOVAs revealed that non-smokers, DS and NDS did not differ in age, sex ratio, BDI score, STAI-trait and STAI-states scores (*p*s > 0.19). As expected, the three groups differed in CO levels [*F*(2,64) = 88.30, *p* < 0.01, $\eta_{p}^{2}$ = 0.62] with non-smokers having lower CO levels than NDS [*t*(43) = -11.06, *p* < 0.01] and DS [*t*(41) = -5.20, *p* < 0.01]. Importantly, DS had lower CO levels than NDS [*t*(40) = -8.06, *p* < 0.01], confirming a successful deprivation manipulation. NDS did not differ from DS with regard to the age when they initiated smoking and the number of years they had smoked (*p*s > 0.06), though they had higher FTND scores [*F*(1,41) = 6.09, *p* < 0.05] and reported more consumption of cigarettes per day [*F*(1,41) = 4.23, *p* < 0.05] than DS.

**Table 1:** Sample characteristics and means scores (and standard deviations) of questionnaires.

Participant characteristics	Non-smokers (NS; *n* = 23)	Non-deprived smokers (NDS; *n* = 22)	Deprived smokers (DS; *n* = 20)
Age (years)	23.35 (2.82)	24.14 (3.30)	25.50 (7.24)
Sex ratio (males/females)	0.92	0.83	1
CO (ppm)	1,17 (1,03)	17,18 (6,86)	4,10 (2,47)
Cigarettes per day	N/A	16,82 (4,22)	13,95 (4,82)
Age to start smoking	N/A	15,73 (2,12)	17,65 (4,12)
Years smoking	N/A	8,41 (3,69)	7,85 (4,74)
Fagerström Test for Nicotine Dependence (FTND)	N/A	4,18 (1,68)	2,75 (2,07)
State Trait Anxiety Inventory (STAI)-trait	37,26 (9,75)	35,73 (7,17)	38,80 (9,17)
STAI-state	35,04 (7,00)	33,86 (6,68)	36,95 (11,39)

### Effect of Reappraisal on Emotional Experience

**FIGURE 1:**
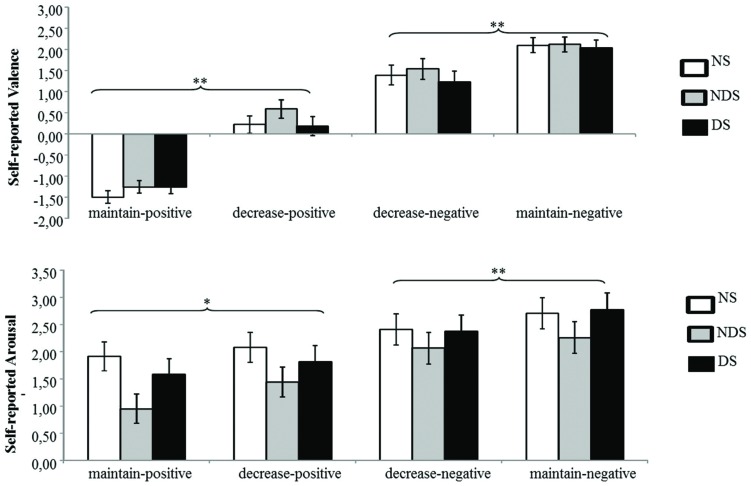
**Mean changes in self-reported valence (top) and arousal (bottom) as a function of reappraisal among non-smokers (NS), non-deprived smokers (NDS), and deprived smokers (DS).** Depicted are difference scores (specific emotion condition minus baseline condition; see Materials and Methods). The more positive difference scores represent more negative **(top)** and more arousing **(bottom)** self-reported emotion under specific emotion condition comparing to baseline condition. Error bars represent standard error of the mean (SEM). ^∗^*p* < 0.05, ^∗∗^*p* < 0.01.

The mean changes in self-reported valence and arousal as a function of reappraisal condition among NS, NDS, and DS are shown in **Figure 1**.

#### Self-reported Valence

The ANOVA revealed main effects of reappraisal [*F*(1,62) = 48.55, *p* < 0.01, $\eta_{p}^{2}$ = 0.44] and picture valence [*F*(1,62) = 238.99, *p* < 0.01, $\eta_{p}^{2}$ = 0.79], and an interaction effect of picture valence by reappraisal [*F*(1,62) = 92.80, *p* < 0.01, $\eta_{p}^{2}$ = 0.60]. Indicating successful regulation of negative and positive emotions via reappraisal, Paired *t*-tests showed that participants reported less negative emotion under the decrease-negative condition compared to the maintain-negative condition [*t*(64) = 5.46, *p* < 0.01], and similarly, less positive emotion under the decrease-positive condition compared to maintain-positive condition [*t*(64) = 11.09, *p* < 0.01]. However, neither the main effect of group nor related interaction effects reached statistical significance (*p* > 0.22). This suggests that all participants successfully down-regulated emotional valence via reappraisal.

#### Self-reported Arousal

The ANOVA revealed a significant main effect of picture valence [*F*(1,62) = 70.40, *p* < 0.01, $\eta_{p}^{2}$ = 0.53] and an interaction effect of picture valence by reappraisal [*F*(1,62) = 18.27, *p* < 0.01, $\eta_{p}^{2}$ = 0.23]. Paired *t*-tests showed that the participants reported less arousal under the decrease-negative condition compared to the maintain-negative condition [*t*(64) = 3.24, *p* < 0.01], but larger arousal under the decrease-positive condition compared to the maintain-positive condition [*t*(64) = 2.13, *p* < 0.05]. Neither the main effect of group nor interaction effects reached statistical significance (*p* > 0.22), indicating that the three groups of participants did not differ in the regulation of emotional arousal via reappraisal.

### Effect of Reappraisal on Psychophysiological Responses

#### Corrugator Activity

**FIGURE 2:**
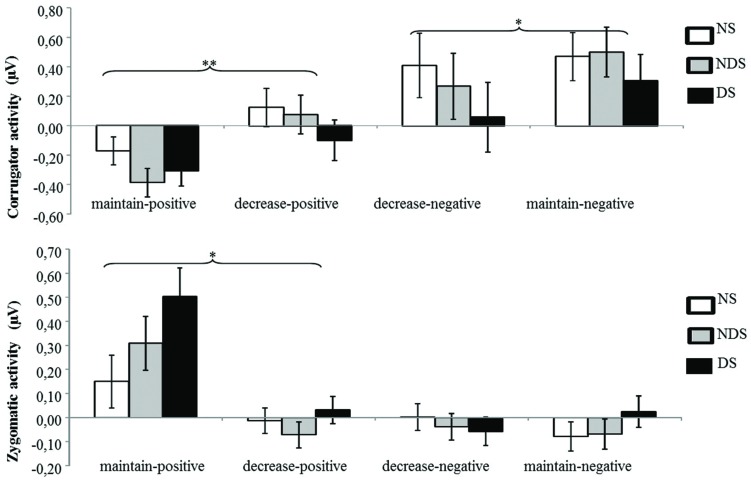
**Mean changes in facial electromyography (EMG) activity as a function of reappraisal among NS, NDS, and DS.** Depicted are difference scores (specific emotion condition minus baseline condition; see Materials and Methods) in corrugator activity **(top)** and zygomaticus activity **(bottom)**. The more positive difference scores in corrugator activity **(top)** represent more negative facial expressions; in contrast, the more positive difference scores in zygomaticus activity **(bottom)** represent more positive facial expressions under specific emotion condition comparing to baseline condition. Error bars represent standard error of the mean (SEM). ^∗^*p* < 0.05, ^∗∗^*p* < 0.01.

The ANOVA revealed a significant main effect of picture valence [*F*(1,62) = 37.56, *p* < 0.01, $\eta_{p}^{2}$= 0.38] and a significant interaction of picture valence by reappraisal [*F*(1,62) = 21.04, *p* < 0.01, $\eta_{p}^{2}$ = 0.25]. Paired *t*-tests showed that the corrugator activity was smaller under the decrease-negative condition compared to the maintain-negative condition [*t*(64) = 2.00, *p* < 0.05], indicating less negative facial expression as a result of reappraisal. Similarly, the corrugator activity was greater under the decrease-positive condition compared to the maintain-positive condition [*t*(64) = 3.74, *p* < 0.01], suggesting a successful down-regulation of positive emotion (see **Figure 2**). Neither the main effect of group nor related interactions reached statistical significance (*p*s > 0.31), indicating that non-smokers, NDS and DS did not differ in the reappraisal outcome on corrugator activity.

#### Zygomaticus Activity

The ANOVA revealed a significant main effect of picture valence [*F*(1,62) = 25.03, *p* < 0.01, $\eta_{p}^{2}$ = 0.29], a main effect of reappraisal [*F*(1,62) = 18.94, *p* < 0.01, $\eta_{p}^{2}$ = 0.23], and a significant interaction of picture valence by reappraisal [*F*(1,62) = 15.11, *p* < 0.01, $\eta_{p}^{2}$ = 0.20]. Paired *t*-tests showed that zygomaticus activity was smaller under the decrease-positive condition compared to the maintain-positive condition [*t*(64) = 4.49, *p* < 0.01], indicating less positive facial expressions as a result of reappraisal (see **Figure 2**). The zygomaticus activity under the maintain-negative condition did not differ from the decrease-negative condition [*t*(64) = 0.35, *p* = 0.73]. Again, neither the main effect of group nor other related interaction effects reached statistical significance (*p*s > 0.16), suggesting similar patterns of emotion regulation among NDS, DS and non-smokers.

#### LPP Activity

**FIGURE 3:**
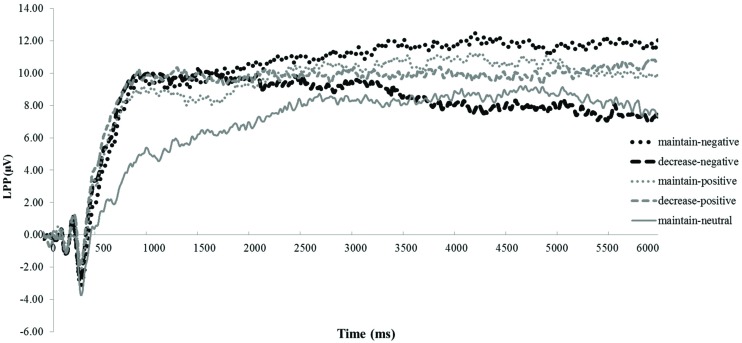
**The time course of late positive potential (LPP) activity.** Depicted are LPP activities in each experimental condition collapsed across groups: maintain-neutral (black dotted line), maintain-negative (black solid line), decrease-negative (gray solid line), maintain-positive (black slashed line), and decrease-positive (gray slashed line).

**FIGURE 4:**
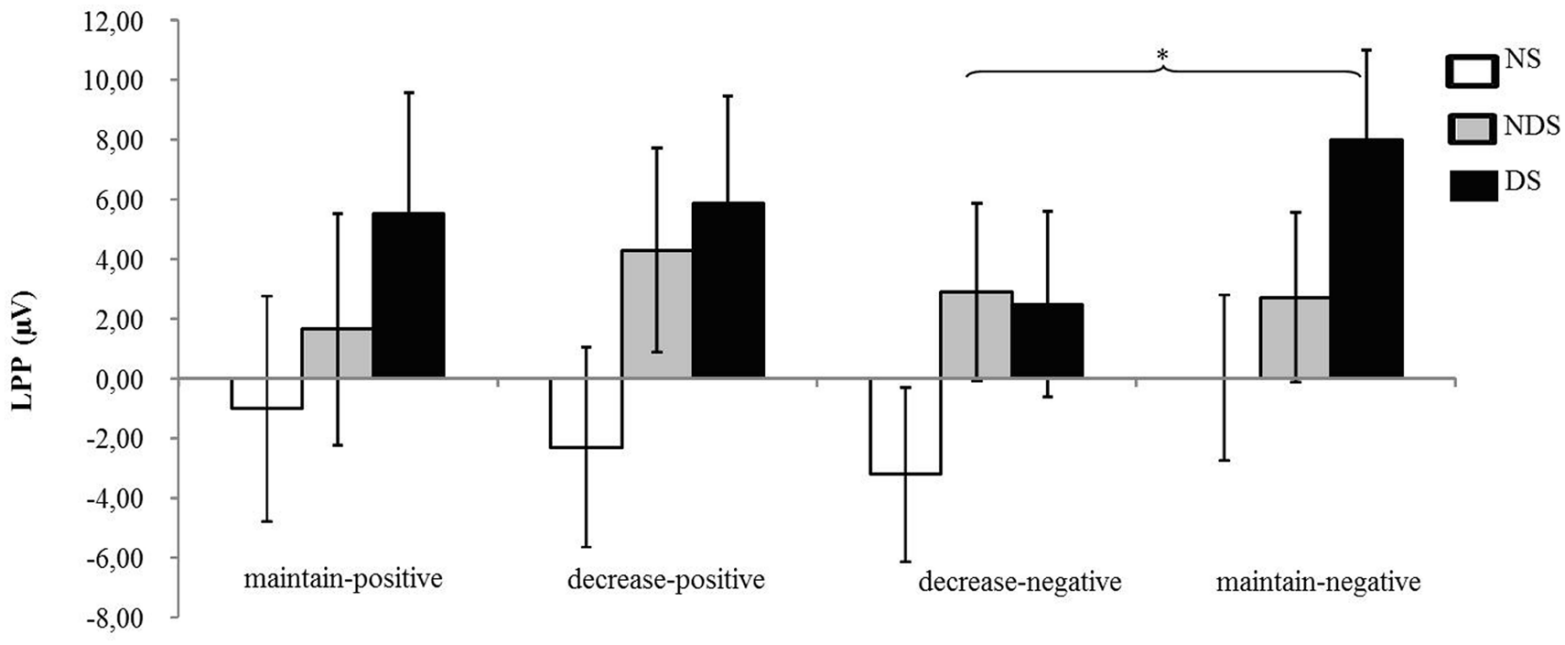
**Mean changes in LPP activity.** Depicted are difference scores (specific condition minus neutral baseline condition; see Materials and Methods) in LPP activity as a function of reappraisal among NS, NDS, and DS. The more positive difference scores represent larger LPP activity under specific emotion condition comparing to baseline condition. Error bars represent standard error of the mean (SEM). ^∗^ *p* < 0.05.

The ANOVA revealed that none of the main or interaction effects reached statistical significance (*p*s > 0.22). However, for explorative purposes, we conducted a paired *t*-tests revealing that the LPP was smaller under the decrease-negative condition compared to the maintain-negative condition [*t*(64) = 2.02, *p* < 0.05], indicating an effect of emotion regulation on the LPP in the expected direction (see **Figures 3** and **4**). However, the difference in LPP activity between the maintain-positive condition and the decrease-positive condition was not significant [*t*(64) = 0.23, *p* = 0.82], suggesting that positive emotion regulation was not reflected in LPP responses.

### Correlation between Changes in Emotions and Changes in Smokers’ Cigarette Craving

**FIGURE 5:**
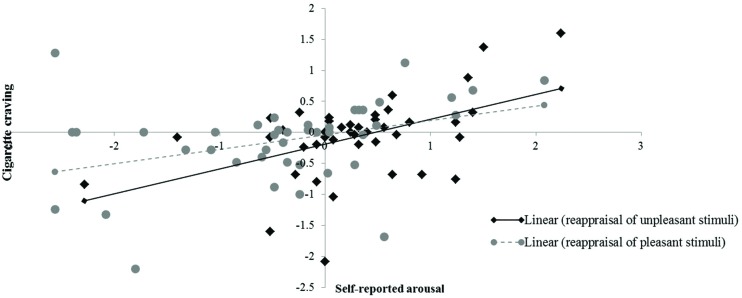
**Correlation between emotional arousal and cigarette craving.** *X*-axis represents changes in self-reported arousal between reappraisal conditions (i.e., decrease-positive, decrease-negative) and corresponding ‘maintain’ conditions (i.e., maintain-positive, maintain-negative). *Y*-axis represents changes in cigarette craving as a function of reappraisal.

Correlation analysis showed that the changes in smokers’ cigarette craving were exclusively correlated with the modulation of self-reported arousal irrespective of the valence of the pictorial stimuli [negative stimuli (*N* = 42; *r* = 0.48, *p* < 0.01), positive stimuli (*N* = 42; *r* = 0.37, *p* < 0.05)]. None of the other correlations reached statistical significance. These correlations reflect that an increase in arousal was associated with an increase in craving (see **Figure 5**).

## Discussion

This study aimed to investigate whether general emotion regulation competence via reappraisal is deteriorated in nicotine addicts. The present study found that all participants were capable of regulating positive and negative emotions following reappraisal instructions in the context of moderately evocative pictures, suggesting that smokers, including NDS and DS, have no deficit in general emotion regulation via deliberate reappraisal.

According to theoretical models of nicotine addiction (e.g., self-medication model and self-regulation failure model), people encounter repeated emotion regulation failures are prone to develop nicotine addiction because they expect that smoking could help them regulate emotions (Baumeister and Heatherton, 1996; Khantzian, 1997; Yucel et al., 2007). Neuroimaging studies have demonstrated that nicotine addicts are associated with abnormal PFC functions that are involved in cognitive emotion regulation (Lubman et al., 2004; Ochsner et al., 2004; McRae et al., 2010; Mocaiber et al., 2011; Moratti et al., 2011; Sutherland et al., 2012). It was assumed that heavy smokers would show general emotion regulation deficits in a reappraisal task. However, our results failed to support this hypothesis. The examined smokers were capable to regulate emotions via deliberate reappraisal, neither in case of positive emotions nor in case of negative emotions. Yet, some characteristics of the emotion regulation task and the experimental stimuli used may explain the inconsistent findings. First, we specifically examined reappraisal as a cognitive emotion regulation strategy and instructed the participants to regulate emotions using this strategy. Second, we used pictorial stimuli with moderate emotion to investigate the general ability of smokers to regulate emotions. This is different from real life situations in which individuals may often experience more intense and arousing stimuli (e.g., smoking related stimuli) and have to decide by themselves when and how to regulate their emotions. Therefore, the present study indicates that heavy smokers may have no deficit in general emotion regulation via reappraisal, which may not exclude emotion regulation dysfunctions in real life situation. Without deliberate instructions, it might be possible that smokers select maladaptive emotion regulation strategies which may result in a failure in regulating emotions (Ehring et al., 2010). To extend this conclusion, future studies are needed to investigate how smokers differ from non-smokers in the spontaneous selection of emotion regulation strategies in a real life situation when presented with more arousing or addiction relevant stimuli.

An additional aim of this study was to investigate the effect of smoking deprivation on general emotion regulation. Prior work showed that DS performed less well than NDS on a variety of cognitive tasks such as attention, memory, and affective processing (Cinciripini et al., 2006; Piper and Curtin, 2006). Thus, we assumed that smoking deprivation may worsen a hypothesized deficit in cognitive emotion regulation. However, our results demonstrated that DS performed as well as NDS when they were instructed to regulate emotions via reappraisal. This suggests that overnight abstinence from smoking does not affect deliberate regulation of emotion in smokers.

The present study expands previous studies by investigating cognitive emotion regulation in terms of both positive and negative stimuli among smokers. It has been noted that regulations of both positive and negative emotions contribute to human well-being and prevent people from substance abuse (Fredrickson, 2001; Fredrickson et al., 2008). However, most emotion regulation research focused on altering negative emotions (Ochsner et al., 2002, 2004; Ochsner and Gross, 2007; McRae et al., 2010; Mocaiber et al., 2011; Parvaz et al., 2012), with a few exceptions that have investigated regulation of positive emotions (Delgado et al., 2008; Giuliani et al., 2008; Krompinger et al., 2008; Wu et al., 2012). Overall, there is a lack of information on the regulation of positive emotions in nicotine addicts. The current findings showed that reappraisal is an efficient way for smokers and non-smokers to regulate both positive and negative emotions, with the outcomes of positive emotion regulation were somewhat different from the ones of negative emotion regulation. Specifically, both smokers and non-smokers successfully reduced negative emotions as indexed by self-ratings of unpleasantness, experienced arousal, corrugator activity, and LPPs. With respect to positive emotions, participants successfully decreased self-reported pleasantness and zygomatic activity, but increased self-reported arousal and failed to change LPPs. These results suggest that changes of emotional valence and arousal as a function of reappraisal are congruent in the context of negative picture stimuli but incongruent in the context of positive picture stimuli. In line with this, previous studies have been demonstrated that more negative stimuli were consistently rated as more arousing, whereas the more positive stimuli were associated with either higher arousal ratings or lower arousal ratings (Lang et al., 2005). Therefore, it should be cautious for future studies to differentiate valence and arousal when addressing regulation of positive emotions.

This study is the first to address the correlation between the effects of reappraisal on emotional valence, arousal and craving in smokers. Previous studies indicated emotion regulation and craving regulation activate common brain regions (Koob and Volkow, 2010). Accordingly, it was assumed that emotions and cravings would be altered simultaneously by reappraisal. The present study showed that smokers’ cigarette craving is positively correlated with emotional arousal with regard to both the negative and the positive stimuli. This expands our understanding of an association between emotions and craving, i.e., arousing stimuli or scenarios may trigger cigarette craving in smokers irrespective of their valence (Velicer et al., 1990; Shiffman et al., 2012). Therefore, it might be plausible to conclude that cigarette craving is linked to emotional arousal rather than emotional valence.

Finally, there are some limitations of this study. First, emotional events in real-life situations could be more intensive than the pictorial stimuli used in the present study. The present study showed that smokers might have an intact ability to regulate emotions via reappraisal, although this does not exclude an inability to select and apply adaptive strategies to regulate emotions in real-life situations, and, in particular, to regulate the motivational responses to smoking related stimuli. Second, the focus of this study was constrained on smokers who do not have a personal history of drug addiction excluding nicotine dependence and do not have current psychiatric or neurological disorders. Those smokers performed as well as non-smokers in the emotion regulation task. To expand this conclusion, future studies are needed to investigate emotion regulation in smokers with comorbid psychiatric disorder.

In sum, the current study illustrates that heavy smokers are able to regulate emotion via deliberate reappraisal, irrespective of the valence of the emotional stimuli. Moreover, we found no indication that over-night deprivation from smoking does affect the performance in the deliberate reappraisal task. From these results, we suppose that heavy smokers do not have a cognitive impairment in general emotion regulation via deliberate reappraisal, although this does not exclude their inability to select and apply appraisal strategies to regulate emotions in real-life situations.

## Conflict of Interest Statement

The authors declare that the research was conducted in the absence of any commercial or financial relationships that could be construed as a potential conflict of interest.

## Footnotes

1 Neutral pictures: 2102 2393 2575 2580 2593 5530 5740 7002 7004 7010 7056 7090 7130 7140 7150 7175 7211 7217 7491 7500 7550 7595 7700 7705 7950; Negative pictures: 1050 1201 1302 1930 2120 2130 2141 2205 2399 2661 2683 2688 2691 2700 2710 2716 2750 2810 3168 3220 3301 6020 6190 6212 6250 6312 6313 6570 6571 6830 6831 8230 9042 9050 9250 9400 9421 9425 9470 9490 9520 9584 9600 9611 9635 9800 9901 9911 9920 9921; Positive pictures: 1463 1710 1811 2080 2150 2160 2340 2345 2352 2550 2655 4572 4608 4623 4660 5270 5300 5450 5460 5480 5600 5623 5626 5628 5629 5660 5700 5910 7501 7502 8030 8034 8040 8080 8090 8116 8117 8161 8170 8180 8190 8200 8210 8300 8370 8400 8490 8496 8502 8500.
